# Prevalence of Forward head posture among car and bike drivers and its relation with neck and cardiopulmonary health parameters- a cross-sectional study

**DOI:** 10.1371/journal.pone.0307016

**Published:** 2024-08-08

**Authors:** Aafreen Aafreen, Abdur Raheem Khan, Ashfaque Khan, Ausaf Ahmad, Mohammad Abu Shaphe, Mohammed M. Alshehri, Ramzi Abdu Alajam, Ali Hakamy, Abdulfattah S. Alqahtani, Taimul Ali, Monira I. Aldhahi

**Affiliations:** 1 Department of Physiotherapy, Integral University, Lucknow, India; 2 Department of Community Medicine, Integral University, Lucknow, India; 3 Physical Therapy Department, College of Applied Medical Sciences, Jazan University, Jazan, Saudi Arabia; 4 Faculty of Applied Medical Sciences, Respiratory Therapy Department, Jazan University, Jazan, Saudi Arabia; 5 Department of Rehabilitation Sciences, College of Applied Medical Sciences, King Saud University, Riyadh, Saudi Arabia; 6 Peerless Hospitex Hospital & Research Center, Kolkata, India; 7 Department of Rehabilitation Sciences, College of Health and Rehabilitation Sciences, Princess Nourah bint Abdulrahman University (PNU), Riyadh, Saudi Arabia; The Hong Kong Polytechnic University, HONG KONG

## Abstract

**Objectives:**

This study aimed to evaluate and compare the prevalence of Forward Head Posture (FHP) in car and bike drivers, and its potential correlation with neck and cardiopulmonary parameters.

**Methods:**

This cross-sectional study involved 400 participants from urban and suburban areas around Lucknow, Uttar Pradesh, India, including 200 car drivers and 200 bike drivers aged 18–65 years with a minimum five-year driving history. Neck health was assessed using measurements such as cervical range of motion and Neck Disability Index (NDI), cardiopulmonary parameters were evaluated through resting heart rate, blood pressure, and pulmonary function tests using the spirometry test, and FHP was assessed using Surgimap application. Statistical analysis was performed using IBM SPSS Statistics software (version 26.0) and included descriptive statistics, hypothesis testing, Chi-square or Fisher’s exact test for binary data, and correlation analyses.

**Results:**

The result show that difference in the mean FHP between car and bike drivers was statistically significant (p = 0.0001), indicating a higher prevalence of FHP among car drivers than among bike drivers. Correlation analyses revealed significant associations between FHP and neck health metrics, especially cervical flexion (r = 0.71, p<0.05), (r = 0.78, p<0.05) and left-side rotation (r = 0.56, p<0.05), (r = 0.61, p<0.05) in car and bike drivers. Among the cardiopulmonary parameters, significant correlations with FHP were observed in resting heart rate (r = 0.33, p<0.05), (r = 0.42, p<0.05), spirometry results FVC (r = 0.29, p<0.05), FEV1 (r = 0.22, p<0.05), and FVC (r = 0.31, p<0.05) for car and bike drivers.

**Conclusion:**

We observed a higher incidence of FHP in car drivers, indicating that a prolonged static posture may lead to greater postural deviation than dynamic movement during biking. This association suggests that FHP could have wide-reaching implications for systemic health, beyond musculoskeletal issues. These findings have the potential to influence preventative strategies and interventions aimed at improving the overall health outcomes for drivers.

## Introduction

Forward head posture (FHP) refers to a condition in which the position of the head shifts forward in relation to the shoulders or lower spine rather than being in an optimal position where the ears align with the shoulders [1]. In transportation, the ergonomic design of vehicles, particularly cars, can play a pivotal role in the onset and progression of FHP, given the extended hours that drivers spend in static positions [2].

Specifically, car drivers are susceptible to FHP because of the nature of their seated posture, extended driving periods, and potential stress from traffic and road conditions [3]. However, despite being at risk, bike drivers maintain a more dynamic posture, consistently adjusting their body alignment with the changing terrain and speed [4]. Thus, physical demands and postural dynamics differed considerably between the two cohorts.

A few studies have explored the relationship between FHP and neck health, finding connections between malalignment and reduced range of motion, increased neck disability, and changes in cervical spine radiographs [5,6]. However, its effects on the cardiopulmonary parameters remain unclear. Some evidence suggests that poor dorsal spine postural alignment may negatively impact lung function and cardiovascular efficiency, although research in this area is limited [7,8]. This study is significant because it aims to fill this critical research gap by investigating the broader health implications of FHP, particularly its impact on cardiopulmonary parameter among car and bike drivers.

We chose to focus on car and bike drivers because of their unique occupational and daily exposure to prolonged static or semi-static postures that can exacerbate FHP. This demographic is at an increased risk of developing FHP and its associated health impacts due to the nature of their work and lack of movement diversity. Previous studies have highlighted that the occupational hazards of sedentary work cause increased musculoskeletal disorders. By focusing on drivers, we aimed to investigate the population with an increased risk of FHP, and its potential impact on neck health and cardiopulmonary function. This targeted approach allowed us to address the existing gap in research regarding the car and bike driving impact on postural health and extend it to include cardiopulmonary parameters.

Although interventions such as ergonomic driving adjustments and physical therapy exercises have been proposed to mitigate the effects of FHP [9], they have limitations. Notably, adherence to these recommendations can be inconsistent, and the effectiveness of such interventions is contingent on the degree of FHP severity, duration, and other individual factors [10]. Therefore, a holistic understanding of FHP and its broader health implications is crucial. This study aimed to fill the critical gap in existing research by providing comprehensive insights into the prevalence of FHP among car and bike drivers, its association with neck health, and its potential impact on cardiorespiratory function. The rationale for this research is grounded in the observation that while the musculoskeletal consequences of FHP are increasingly recognized, its effects on cardiorespiratory health are not well understood. Addressing this gap is important for developing targeted interventions and informing policy decisions that can mitigate health risks associated with FHP in urban commuting populations.

This study aimed to provide a deeper understanding of the prevalence of FHP in car and bike drivers and to determine any potential correlations among the severity of FHP, neck health, and cardiopulmonary parameters. Using a cross-sectional design and examining 400 participants, the study sought to offer comprehensive insights into the prevalence of FHP, its association with neck health, and its potential impact on cardiorespiratory function. This research is crucial for developing targeted interventions and informing policy decisions that can mitigate health risks associated with FHP in urban commuting populations.

The modern world continues to grow with technological advancements as well as the challenges it presents to human health. The impact of FHP on drivers, both cars and bikes, requires attention not only from a musculoskeletal perspective, but also from a cardiopulmonary lens. By embarking on this exploration, this study aimed to offer insights that could provide a strong direction for urban commuters to aligns with both technological progress and human health priorities. By integrating ergonomic solutions and wellness initiatives into urban planning and driver education programs, we can foster environments conducive to improved posture and overall well-being.

## Material and methods

### Design, setting and population

In this cross-sectional study conducted from December 2022 to June 2023, 400 participants were selected from urban and suburban settings, local car and bike driving communities, associations, and clubs near Integral University, Lucknow. This group was equally split into 200 car drivers and 200 bike drivers. The participants were aged between 18 and 65 years and had a driving history of at least 5 years. They were individuals who primarily drove either a car or a bike, with minimal overlap between the two modes. On the other hand, individuals were excluded if they had neck trauma, surgery, any underlying neurological or systemic conditions affecting the neck, pregnancy, an inability to comprehend or respond to study assessments, any known respiratory or cardiovascular conditions that might skew results, were professional athletes, had occupations significantly influencing posture (e.g., ballet dancers, manual labourers), or were undergoing current physical therapy or ergonomic intervention for FHP.

Stratified random sampling was implemented to ensure a balanced representation of urban and suburban areas, with each stratum meticulously assembled to include an even distribution of both driver categories.

The sample size for the study was determined using the following formula, *n* = *z*^2^ *p*(1 − *p*)/*d*^2^, where, p = 50%, confidence level 95%, Z score = 1.96, and margin of error (d) = 5%, comprising 400 study samples.

Before embarking on the study, all participant were briefed about the objectives of the study, and provided written informed consent. This study adhered to the ethical principles of the Declaration of Helsinki (World Medical Association, 2013). Ethical clearance was granted by the Institutional Ethical Committee (IEC/IIMS&R/2022/70) of the Integral University, Lucknow, India. The study was registered under the Clinical Trial Registry India (CTRI) with registration number CTRI/2022/11/047689.

### Procedure

#### Neck health assessment

We used multiple instruments for a comprehensive neck health assessment. The Cervical Range of Motion (CROM) was determined using a smartphone-based clinometer and compass application, and the cervical active range of motion in flexion and extension, right and left lateral flexion, and right and left rotation was measured for each participant in both groups. Before measurement, the participants removed any eyeglasses or hats and lifted and fastened any hair covering the ears, neck, or eyes. They observed a brief demonstration of the six cervical motions to be performed. Cervical range of motion was measured in a standard sitting position to remove errors, and two smartphone (iPhone) apps were used. The smartphone app used to measure the cervical range of motion in the frontal and sagittal planes were Clinometer (Peter Breitling, Version 3.3, http://www.plaincode.com/products), an app designed using the three inbuilt accelerometers (LIS302DL accelerometer). This app uses internal three-axis linear accelerometers to measure the direction of the gravity pull. For this, the gyroscope remains in one position, regardless of the orientation. When placed against a solid surface, the inclinometer compares the angle of the object to that of the gyroscope and displays the results using the software interface. The smartphone applications demonstrated a high level of reliability, as indicated by their intraclass correlation coefficient (ICC) of 0.80. In comparison to a universal goniometer, these applications presented good to excellent validity, as indicated by their intraclass correlation coefficients of 0.65, for all six cervical ranges of motion [11].

The Neck Disability Index (NDI) is a ten-item questionnaire that measures the disability caused by whiplash and neck pain and is based on the Oswestry Low Back Pain Index. There are six measures pertaining to activities of daily life: lifting, working, driving, having fun, taking care of one person, and reading. Four measures were related to subjective symptomatology: pain intensity, headache, concentration, and sleep. The administration of the questionnaire requires no additional training and only takes 5–10 min to complete and score. It is important to note that previous research has demonstrated good reliability and validity of the NDI, making it a reliable tool for assessing disability related to neck pain [12].

Participant Instructions and Scoring: Each question had six possible responses, ranging from no disability (zero) to entire impairment (six) (5). The overall score ranges from 0 (no disability) to 50, when the 10 components are added together (maximum disability). This rating was expressed as a percentage. A score of less than 4 denotes no disability, a score of 5–14 indicates light disability, a score of 15–24 indicates moderate disability, a score of 25–34 indicates severe disability, and a score of more than 35 denotes complete disability [13].

#### Cardiopulmonary parameters evaluation

Cardiopulmonary function was evaluated through the use of an automated monitor to measure resting heart rate and blood pressure, and spirometry to assess respiratory parameters in accordance with the guidelines established by the American Thoracic Society [14–16].

#### Forward Head Posture (FHP) assessment

For assessing FHP, the craniovertebral angle was measured across different age groups and postures using a smartphone application [17]. Craniovertebral angle (CVA) was gauged using Surgimap, specialized software for evaluating spinal alignment and posture. Participants were side-photographed in their natural stance, with Surgimap analysing the cervical spine’s horizontal alignment; quantifying the craniovertebral angle (CVA). A smaller CVA implies a more pronounced forward head posture (FHP). An image of the sagittal plane of each subject was captured with objective access to the CVA, and the digital camera was positioned at 1.5 m and fixed with a camera stand without any rotation or tilt. To standardize the subject’s head and neck position, the level of the camera was set to the height of the subject’s shoulder, and the obtained image was analysed using Surgimap system software. If an individual’s CVA is less than 48°, they are classified as having FHP, whereas a CVA greater than 48° indicates normal craniovertebral posture. It has been stated that this procedure is reliable (ICC = 0.88) [18,19].

#### Procedural framework

Each participant underwent exhaustive physical examination. This was followed by dedicated assessments of CROM, FHP, and cardiopulmonary parameters. All assessments were conducted by experienced and skilled physiotherapists, and all readings for each participant were recorded on the same day and at the same location to minimize variations in environmental conditions and participant readiness, thereby enhancing the reliability and validity of the results.

Although the assessment process was standardized, the car and bike drivers were evaluated separately. This decision was aimed at optimizing the effectiveness and accuracy of the assessment process to better serve both car and bike drivers.

### Statistical analysis

IBM SPSS Statistics software (version 26.0) was used for statistical analysis. Descriptive statistics were calculated separately for car and bike drivers for each variable, including means, standard deviations, medians, and interquartile ranges. Hypothesis testing was performed to compare the prevalence of FHP between car and bicycle drivers. Chi-square test or Fisher’s exact test was used for binary data (FHP prevalence).

Correlation analyses were conducted to separately examine the relationships among FHP prevalence, neck health, and cardiopulmonary parameters for car and bike drivers. Appropriate correlation coefficients (e.g., Pearson’s correlation and Spearman’s rank correlation) are based on the distribution of the data.

## Results

### Participant demographics

A total of 400 car and bike drivers aged between 18 and 65 years were enrolled, and their data were recorded. Table 1 provides participant demographics categorized by mode of transportation, distinguishing between car and bike drivers. The mean age of the overall group was 37.5 years with a standard deviation of 9.2. When comparing car drivers to bike drivers, the mean age slightly differs, with car drivers having an average age of 38.4 years (±8.6) and bike drivers averaging 36.9 years (±8.3). In terms of gender distribution, the overall group consisted of 290 males and 110 females, evenly divided between car and bike drivers (145 males and 55 females in each category). Participants across both groups share a driving experience of 5 years or more, with the mean driving experience being 12.0 years (±5.1) for the overall group. Car drivers exhibited a slightly longer mean driving experience of 12.6 years (±5.2), whereas bike drivers had a mean driving experience of 11.4 years (±4.7). Additionally, the average weekly driving hours for all participants is 17.3 hours (±5.8), with car drivers spending an average of 18.4 hours (±5.9) per week on the road, and bike drivers averaging 16.2 hours (±5.5) weekly.

**Table 1:** Participant demographics.

Variable	Overall	Car Drivers	Bike Drivers
Age Range	18–65 years	18–65 years	18–65 years
Mean Age	37.5± 9.2	38.4± 8.6	36.9 ±8.3
Sex (Male/Female)	290 / 110	145 / 55	145 / 55
Driving Experience Range	5+ years	5+ years	5+ years
Mean Driving Experience	12.0 ±5.1	12.6 ±5.2	11.4 ±4.7
Average Weekly Driving Hours	17.3 ±5.8	18.4 ±5.9	16.2 ±5.5

### Neck health and cardiopulmonary parameters assessment

Table 2 presents physical and health-related parameters for both car drivers and bike drivers. The prevalence of Forward Head Posture (FHP) is reported as a percentage, with car drivers exhibiting a prevalence of 30% (±5%), while bike drivers’ show a slightly lower prevalence of 20% (±6%). In terms of cervical mobility, bike drivers generally have slightly higher degrees of cervical flexion (45.7° ± 7.8) compared to car drivers (43.7° ± 7.6). Similarly, bike drivers demonstrate increased cervical extension, right and left side rotation, right and left side flexion when compared to car drivers. The Neck Health Questionnaire Score is noticeably higher for bike drivers (15 ± 3) compared to car drivers (8 ± 2), indicating potential differences in self-reported neck health. Resting heart rate is slightly higher for bike drivers (78 ± 11) than for car drivers (75 ± 10). Blood pressure is within the normal range for both groups, with bike drivers having slightly elevated values (125/85 ± 12/6) compared to car drivers (120/80 ± 10/5). FVC, is reported as 4.5 litres (±0.5) for car drivers and 4.2 litres (±0.6) for bike drivers. FEV1, is reported as 3.5 litres (±0.5) for car drivers and 3.2 litres (±0.6) for bike drivers. FEV1/FVC, is reported as 0.78 (±0.05) for car drivers and 0.76 (±0.06) for bike drivers. These results suggest relatively comparable respiratory function between the two groups, with car and bike drivers demonstrating similar spirometric values.

**Table 2:** Neck health and cardiopulmonary parameters assessment.

Variable	Car Drivers (n = 200)	Bike Drivers (n = 200)	P value
FHP Prevalence (%)	30% ± 5%	20% ± 6%	0.0001
Cervical Flexion (degrees)	43.7 ± 7.6	45.7 ± 7.8	0.0097
Cervical Extension	75.8 ± 4.3	78.0 ± 3.3	0.0001
Right side Rotation	58.6 ± 6.2	63.8 ± 7.7	0.0001
Left side Rotation	57.3 ± 5.4	61.2 ± 4.6	0.0001
Right side flexion	43.7 ± 4.2	47.0 ± 4.6	0.0001
Left side flexion	43.4 ± 5.4	46.7 ± 4.2	0.0001
Neck Health Questionnaire Score	8 ± 2	15 ± 3	0.0001
Resting Heart Rate (bpm)	75 ± 10	78 ± 11	0.0045
Blood Pressure (mmHg) Systolic	120 ± 10	125 ± 12	0.0001
Blood Pressure (mmHg) Diastolic	80 ± 5	85 ± 6	0.0001
FVC	4.5L ± 0.5	4.2L ± 0.6	0.0001
FEV1	3.5L ± 0.5	3.2L ± 0.6	0.0001
FEV1/FVC	0.78 ± 0.05	0.76 ± 0.06	0.0003

**FHP**: Forward Head Posture; **FVC**: Forced Vital Capacity; **FEV1**: Forced Expiratory Volume in the first second.

Table 3 compares the prevalence of FHP between car and bike drivers. Car drivers exhibit a higher FHP prevalence of 30% (±5%) than bike drivers, who had a slightly lower prevalence of 20% (±6%). The difference in FHP prevalence between the two groups was statistically significant (p<0.05). In car drivers, the correlation is reported as (r = 0.53, p<0.05) while for bike drivers, it was slightly higher at (r = 0.56, p<0.05). Both correlations were statistically significant (p = 0.034).

**Table 3:** Comparison of FHP Prevalence and its correlations with neck health questionnaire score in car and bike drives.

	Car Drivers (n = 200)	Bike Drivers (n = 200)	p-value
FHP Prevalence (%)	30% ± 5%	20% ± 6%	<0.05
Neck Health Questionnaire Score	0.53*	0.56*	

**FHP**: Forward Head Posture.

* Statistically significant correlation at p< 0.05.

Table 4 illustrates the correlation coefficients between FHP prevalence and various neck health metrics for both car and bike drivers. In car drivers, a statistically significant positive correlation was observed between FHP prevalence and cervical flexion (r = 0.71, p<0.05), right side rotation (r = 0.66, p<0.05), left side rotation (r = 0.56, p<0.05), right side flexion (r = 0.49, p<0.05), left side flexion (r = 0.44, p<0.05), and Neck Disability Index (NDI) (r = 0.56, p<0.05). Similarly, for bike drivers, there were statistically significant positive correlations between FHP prevalence and cervical flexion (r = 0.78, p<0.05), cervical extension (r = 0.82, p<0.05), right side rotation (r = 0.75, p<0.05), left side rotation (r = 0.61, p<0.05), and NDI (r = 0.53, p<0.05). These findings show that, as FHP prevalence increases, there is a corresponding positive correlation with specific neck health parameters, emphasizing the impact of FHP on various aspects of neck health.

**Table 4:** Correlation between FHP prevalence with neck health metrics for car and bike drives.

	Car Drivers (n = 200)	Bike Drivers (n = 200)
Cervical Flexion (degrees)	0.71*	0.78*
Cervical Extension	0.81	0.82
Right side Rotation	0.66	0.75
Left side Rotation	0.56*	0.61*
Right side flexion	0.49	0.38
Left side flexion	0.44*	0.37*
NDI	0.60*	0.70*

**NDI**: Neck Disability Index.

* statistically significant correlation at p< 0.05.

Table 5 outlines the correlation coefficients between FHP prevalence and cardiopulmonary parameters for both car and bike drivers. In car drivers, statistically moderate positive correlations were observed between FHP prevalence and resting heart rate (r = 0.33, p<0.05), blood pressure (r = 0.56, p<0.05), spirometry results such as FVC (r = 0.29, p<0.05), and FEV1 (r = 0.22, p<0.05) shows mild positive correlation. For bike drivers, statistically significant moderate positive correlations existed between FHP prevalence and resting heart rate (r = 0.42, p<0.05) and, blood pressure (r = 0.61, p<0.05), and spirometry results such as FVC (r = 0.31, p<0.05) showed a mild positive correlation. These findings indicate that, as FHP prevalence increases, there is a corresponding positive correlation with certain cardiopulmonary parameters in both car and bike drivers. The statistically significant correlations highlight the potential influence of FHP on cardiovascular and respiratory parameters.

**Table 5:** Correlation between FHP prevalence with cardiopulmonary parameters for car and bike drives.

	Car Drivers (n = 200)	Bike Drivers (n = 200)
Resting Heart Rate (bpm)	0.33*	0.42*
Blood Pressure (mmHg)	0.56	0.61
Spirometry Results (e.g., FVC)	0.29*	0.31*
FEV1	0.22*	0.12*
FEV1/FVC	0.27	0.16

**FVC**: Forced Vital Capacity; **FEV1**: Forced Expiratory Volume in the first second.

* statistically significant correlation at p< 0.05.

## Discussion

Our study elucidated the prevalence of Forward Head Posture (FHP) among car and bike drivers, each presenting distinct ergonomic challenges that can influence posture over extended periods. A marked observation from our findings is the higher prevalence of FHP in car drivers than in their bike-driving counterparts. This could be ascribed predominantly to the more stationary and sedentary nature of car driving relative to the active position inherent in biking [20].

The methodologies employed were meticulously chosen and appear to have been effective in yielding insightful data. The decision to use smartphone applications, validated in prior studies, for gauging Cervical Range of Motion (CROM) and FHP was proven to be judicious, as it provided precise measurements [11,17]. Furthermore, our inclusion of the Neck Disability Index (NDI) for assessing neck health adds another layer of reliability, given that it has a robust track record in the literature for its reliability and validity [21].

Drawing parallel to the extant literature, the work of Smith et al. resonates with our observations. They identified that individuals in professions that require prolonged sedentary postures, much like car drivers, demonstrated an augmented prevalence of FHP [19,22]. This aligns seamlessly with our study, further reinforcing the hypothesis that prolonged static postures can act as catalysts for the development of FHP [16]. Conversely, biking, which require frequent neck movement and an upright spine, might serve as a protective factor against FHP. This notion is supported by study by Soares, et al., which highlighted diminished FHP incidence among individuals in roles demanding regular motion [23].

However, it’s worth noting that not all literature concurs with our observations. For instance, Ahmadipoor, et al. reported minimal differences in FHP prevalence between individuals in static and dynamic professions [24]. Such disparities in findings might stem from diverse assessment methodologies or demographic variations in the study cohorts. Our study provides a comprehensive view that aligns with the majority of existing literature, supporting the notion of increased FHP prevalence among car drivers [13,19].

Despite the valuable insights generated by this study regarding the connection between neck health and cardiopulmonary parameters among car and bike drivers, certain limitations should be noted. The cross-sectional design inherently leads to challenges when considering causative associations. This design captures a snapshot in time, preventing the establishment of temporal or causal links with certainty. Our study is limited in that it relies solely on the NDI and cervical range of motion as primary outcomes for cervical assessment, as recent literature suggests that psychological well-being and social factors can significantly influence cervical health outcomes. A more comprehensive evaluation approach that consider physical, neurological, psychosocial, and functional aspects is necessary. This can be achieved using advanced imaging techniques, neurophysiological assessments, and questionnaires that capture the psychosocial aspects of neck disorders. Another potential issue is dependence on self-reported driving histories, which might carry inherent biases, although we anticipate their influence on overarching conclusions to be minimal.

Although some studies support our conclusions, others have contrasting views. It is important to understand that such discrepancies might arise because of differences in study designs, methodologies, or demographics among studies. Taking a comprehensive view, it is evident that our study makes a meaningful contribution to the ongoing discussion on FHP, emphasizing its prevalence among specific driver categories.

To express this, our research underscores a call for targeted ergonomic interventions, particularly for car drivers, to mitigate the progression of FHP. Implementing such strategies could play a pivotal role in enhancing an individual’s well-being and preventing potential musculoskeletal complications. For a more conclusive understanding, subsequent longitudinal studies may be invaluable.

Given the scope of our study, we recommend further research to explore causal relationships between FHP and other health parameters. Future studies should consider the development and evaluation of ergonomic and postural interventions tailored to the needs of individuals engaged in activities prone to promote FHP, such as car driving. These interventions, which were not directly investigated in our study, appear to be logical extensions of our findings, and are aimed at improving overall health and well-being.

## Conclusion

Our cross-sectional study elucidated the relationship between Forward Head Posture (FHP) and its impact on neck health and cardiopulmonary parameters in car and bike drivers. We found a significantly higher prevalence of FHP in car drivers, suggesting that static posture associated with prolonged periods of driving may contribute to postural deviations more than dynamic posture associated with biking. Furthermore, the identification of significant correlations among FHP, neck health, and cardiopulmonary parameters, including resting heart rate and spirometry results, highlights the potential of FHP to affect systemic health beyond musculoskeletal discomfort.

Our findings have several implications. First, they reinforced the importance of considering the impact of daily activities and profession on postural health. Second, they suggested that interventions aimed at mitigating FHP could have benefits extending beyond the musculoskeletal system and potentially improving cardiopulmonary function.

Our research emphasizes the significance of ergonomic interventions for car and bike drivers, as forward head posture (FHP) is closely linked to neck discomfort and potential cardiopulmonary consequences. Incorporating regular posture assessments and tailored exercises into driver wellness programs can alleviate health risks and promote overall driver wellness. These initiatives have the potential to improve road safety and public health.

## Supporting information

S1 Checklist (DOCX)

S1 Data (XLSX)
